# Arthrogryposis multiplex congenita with maxillofacial involvement: a case report

**DOI:** 10.1186/s40902-023-00378-6

**Published:** 2023-02-08

**Authors:** Stefano Cirillo, Daniele Regge, Umberto Garagiola, Alessandro Tortarolo, Giuseppe Carlo Iorio, Orges Spahiu, Maria Grazia Piancino

**Affiliations:** 1 Department of Radiology, Mauriziano Umberto I Hospital, Turin, Italy; 2 Department of Surgical Sciences, University of Turin, Turin, Italy; 3 Department of Biomedical Surgical and Dental Sciences Maxillo-Facial and Odontostomatology Unit, School of Orthodontics Fondazione IRCCS Ca’ Granda Ospedale Maggiore Policlinico, University of Milan, Milan, Italy; 4 Department of Surgical Sciences, Dental School, University of Turin, Turin, Italy; 5 Department of Oncology, University of Turin, Turin, Italy

**Keywords:** Congenital multiple arthrogryposis, Musculoskeletal abnormality, Spiral computed tomography, Imaging magnetic resonance, Malocclusion, Temporomandibular joint, Craniomandibular disorder, Function generating bite

## Abstract

**Background:**

Arthrogryposis multiplex congenita is a rare condition that mainly involves the lower limbs, characterized by severe joint deformity and contracture, muscular atrophy, and functional impairment. Its clinical manifestations are heterogenous and may involve the maxillofacial district as well.

**Case presentation:**

This case report describes a 20-year-old patient with arthrogryposis multiplex congenita with skeletal crossbite, facial asymmetry, reduced mouth opening and absence of lateral mandibular movement on the left side. After clinical evaluation, the following exams were required: postero-anterior cephalometric tracing, head and neck electromyography, computerized axiography, computed tomography scan, and maxillofacial magnetic resonance imaging. Orthognathodontic evaluation indicated skeletal asymmetry, reduced condylar movements on the left side and abnormally low electromyography activity of the masticatory muscles on the left side. Computed tomography and magnetic resonance imaging revealed unilateral left mandibular hypoplasia, hypotrophy, and fatty infiltration of masticatory muscles on the left side, as well as immobility of the left condyle during mouth opening, and hypoplasia of the left articular disk, which was however not displaced. Surgery was not indicated and conservative orthognathodontic treatment with function generating bite was suggested to balance the occlusal plane, as well as stretching exercises.

**Conclusions:**

A rare case of arthrogryposis multiplex congenita with maxillofacial involvement illustrates that a patient-centred, multidisciplinary approach with accurate diagnosis is required to formulate the best treatment plan. Because of the considerable damage to the masticatory muscles, conservative orthognathodontic therapy may be the best treatment option.

## Background

Arthrogryposis multiplex congenita (AMC) is a rare form of arthrogryposis, characterized by congenital, non-progressive, and symmetric joint contractures that involve at least two different body areas; both upper and lower limbs are usually involved [1]. It is a heterogeneous condition, with many different forms described in the literature [2]. In this disease, severe limitation of joint motion is frequently associated with a vicious attitude in flexion or hyperextension. As time goes on, deformities with contractures and dislocations worsen, accompanied by atrophy or absence of muscular mass [3].

The incidence of AMC is variably reported to be between 1/3000 and 1/10000 births. Statistical variability is due to the different criteria adopted in defining the disease, which may be easily confused with other syndromes. Involvement of the cranium and maxillofacial region has been observed in 22–24% of cases of arthrogryposis [4], and a reduction of mandibular opening has been observed in 13–25% of those cases [5].

AMC usually occurs sporadically, but different patterns of inheritance have been described, as well as more than 200 genes associated with this disease; however, the molecular mechanism of AMC is not known [2]. Arthrogryposis may be determined by a reduction of articular mobility during intrauterine life (fetal crowding), general connective tissue disorders, neurological disorders, myopathy, intrauterine vascular accidents, or changes in fetal environment (oligohydramnios) [6]. Reduction and fatty replacement of the muscular mass is frequently encountered in this syndrome, and changes of the corresponding spinal metameres, along with reduced anterior horn cell size and number, have been described [7].

## Case presentation

A 20-year-old patient with AMC was referred to the Orthodontics Department, University of Turin, for temporomandibular joint (TMJ) surgical evaluation. Informed consent was obtained from the patient.

Prior to TMJ evaluation, the patient was diagnosed with AMC, involving mainly the lower limbs and the maxillofacial district, with severe mandibular hypomobility. The patient had undergone different surgical procedures, including right knee extension, left hip extension and left achillotenotomy, followed by cycles of physiotherapy and functional re-education. These treatments were partially successful in correcting the lower limb deformities.

At visual inspection, the patient showed macroscopic facial asymmetry, mostly on the left side, and a severe dental and skeletal crossbite of the right hemiarch with relevant functional impairment. Clinical examination showed a reduced maximum opening of the mouth (29 mm) with deviation towards the left side. Lateral excursion of the mandible towards the right side was normal (11 mm), while on the left side it was completely impaired (0 mm).

To investigate the extent of the functional impairment, the following instrumental examinations were performed:

- Postero-anterior (PA) cephalometry, showing a severe mandibular asymmetry with deviation of the medial line towards the right side.
- Computerized axiography, performed with a Condylocomp LR3 (Dentron GmbH, Wurzburg, Germany), indicating that the protrusive and the maximal opening paths showed limitation in extension with a good repeatability on the right side, while on the left side there was total movement blockage.
- Electromyography, performed with an electromyograph K6-I (Myo- Tronics Inc., Seattle, WA-USA). According to Jankelson test I (at rest) an increase of the value of the superior trapezius was observed while the remaining muscles showed physiological values. In test II (maximum clenching) extremely reduced values for the left anterior temporal and masseter muscles were observed and were not modified by the interposition of cotton rolls between the dental arches; the muscles on the right side contracted normally.

**Fig. 1:**
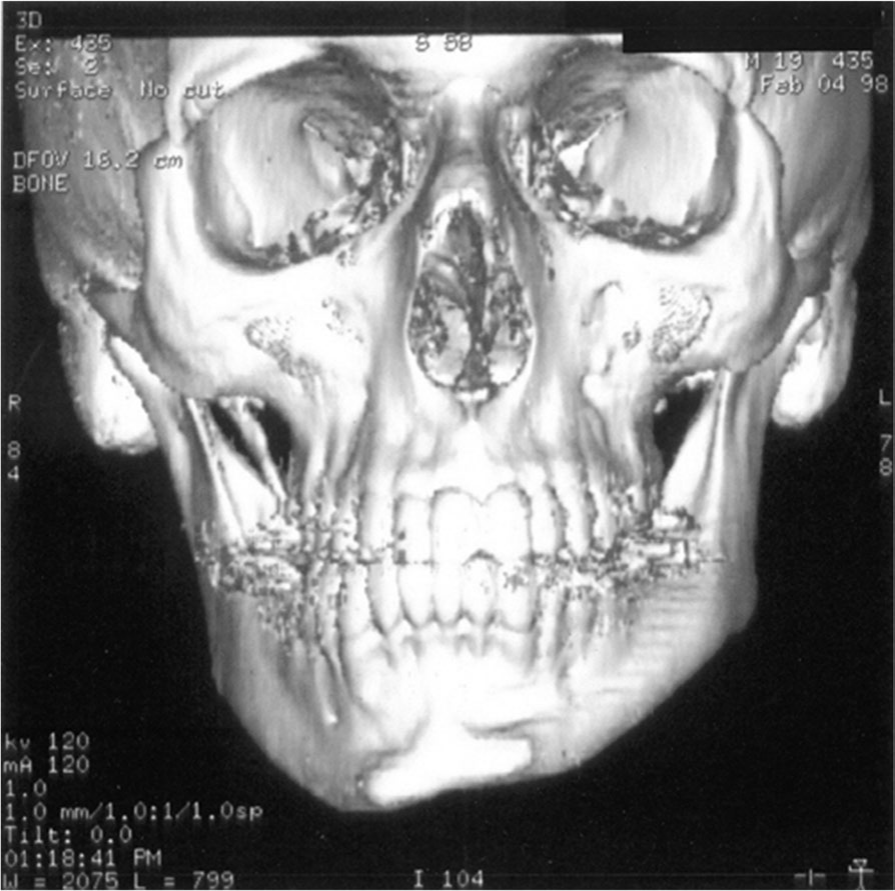
Computed tomography (CT) with 3D reconstruction. The facial asymmetry is due to the underdevelopment of the left side of the mandible

**Fig. 2:**
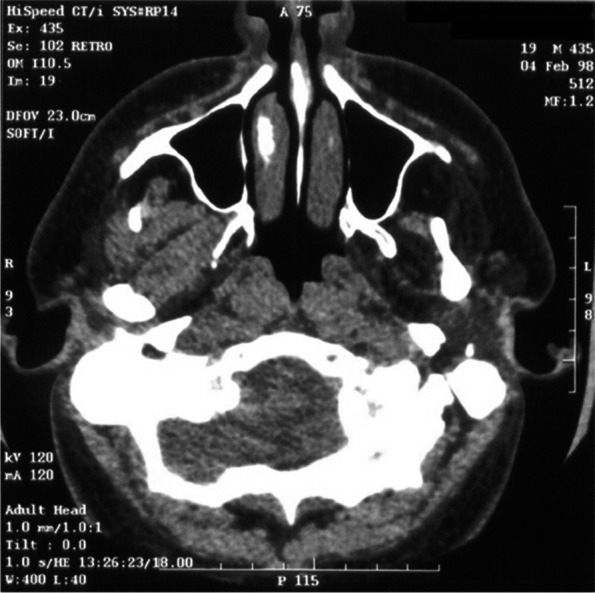
CT scans. The severe atrophy with fat substitution of the left internal and external pterygoid, left masseter and left anterior temporalis muscles is evident

**Fig. 3:**
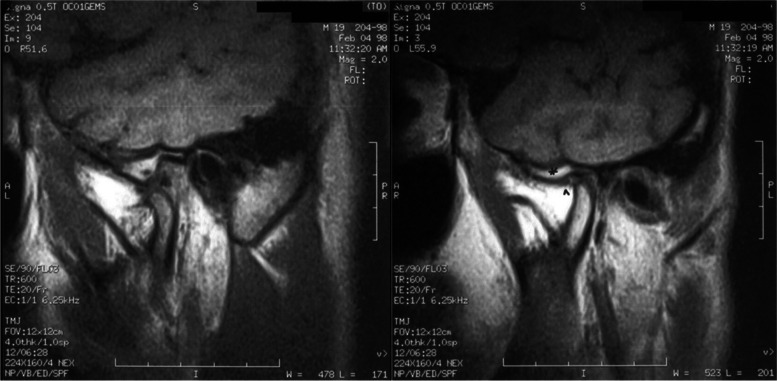
Magnetic resonance imaging (MRI) sagittal T1 weighted scans. **a** right temporomandibular joint (TMJ), open mouth: physiologic joint with normal condylar excursion. **b** left TMJ, open mouth: notice a less pronounced anterior tubercle of the temporal bone and a shallow articular cavity (*); the articular disk is hypoplastic (^); complete condyle immobility is evident

**Fig. 4:**
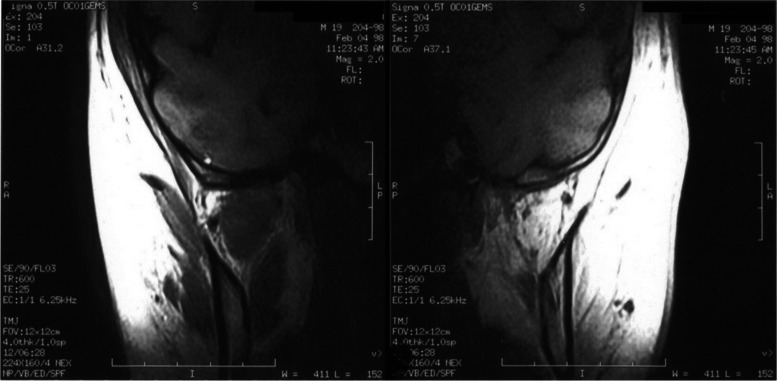
MRI coronal T1-weighted scans. **a** Right side: the masticatory muscles appear normal. **b** Left side: masticatory muscles appear severely hypotrophic

Having found a severe functional impairment on the left side of the mandible, imaging with spiral computed tomography (SCT) and magnetic resonance imaging (MRI) were considered to assess the morphology of the TMJ and the masticatory muscles:

iv) Spiral CT was performed with helical scans in the axial position from the chin tip to the cranium vertex. Slice thickness was 3 mm, reconstruction gap 1 mm. The scans obtained were sent to a Workstation (3.1 Advantage Windows, GE Medical Systems) for 3D reconstruction. Computed tomography (CT) did not show significant anomalies of the condylar structures. On the other hand, significant underdevelopment of the left hemimandible was observed (Fig. 1). Furthermore, there was a serious atrophy with fat substitution of the following muscles: left internal and external pterygoid, left masseter, and left anterior temporalis (Fig. 2).
v) MRI was performed with a Sigma 1.5 MR Unit (General Electric Medical, Milwaukee, Wisc.) to evaluate disk morphology and position, and TMJ mobility. Condylar asymmetry was observed, as the left structure appeared smaller and on a different angle compared to the contralateral. On the left side, anterior tubercle of the temporal bone was less pronounced, the articular cavity was shallow and the coronoid process slightly hypertrophic (Fig. 3). Functional impairment with complete absence of movements of the left condyle during the opening was evident. The articular disk was hypotrophic but it was in a physiological position. There was no disk displacement during mouth opening. MRI confirmed the serious atrophy of the left masticatory muscles, associated with hyperintense signal in the T1-weighted scans due to fatty infiltration (Fig. 4).

The case of AMC described in this paper is of special interest, due to the rarity of the maxillofacial involvement in this disease. The MRI and Spiral CT with 3D reconstruction underlined the value of imaging techniques in the diagnosis and the evaluation of the extent of damage in this disease [8–10]. The treatment of AMC with facial involvement is still controversial. Options include forcible stretching of the hypoplastic masticatory muscles, aimed at improving articular function, and TMJ surgery with bilateral coronoidectomy [11, 12]. Table 1 reports a summary of previously published case-reports of AMC with oral involvement and their treatments [4, 11, 13–16].

**Table 1:** Previously published case-reports of maxillo-facial management of adult AMC patients with TMJ involvement and reduced mouth opening (adapted from Bénard et al. 2021 [13])

Reference	Clinical presentation	Treatment	Outcome
Epstein et Wittenberg, 1987 [11]	Reduced mouth opening; right TMJ pain	Surgical (bilateral coronoidectomy and left condylectomy); physical therapy	Improved mouth opening; reduced pain
Hodgson et al. 1988 [4]	Reduced mouth opening (22mm); no protrusion or lateral excursion	Surgical (left condylectomy); physical therapy	Improved mouth opening (+ 8 mm)
Thomas et al. 2001 [14]	Reduced mouth opening (9mm)	Surgical (bilateral coronoidectomy and meniscectomy, lateral pterygoid myotomy, capsular release); physical therapy	Improved mouth opening (+ 9 mm)
Kargel et al. 2007 [15]	Reduced mouth opening (25mm), anterior open bite	Surgical (bilateral coronoidectomy, orthognathic surgery); orthodontic	Improved mouth opening (+ 6 mm), correction of anterior open bite
Nordone et al. 2010 [16]	Reduced mouth opening (15mm), no protrusion or lateral excursion	Physical therapy; surgical (bilateral coronoidectomy, condylectomy, TMJ arthroplasty and arthrotomy with osteodistraction)	Improved mouth opening (+ 10 mm)
Bénard et al. 2021 [13]	Reduced mouth opening (3mm)	Surgical (bilateral coronoidectomy and TMJ arthroplasty); physical therapy	Improved mouth opening (+ 14 mm)

*TMJ* temporomandibular joint

To assess the most appropriate therapeutical strategy, the patient was evaluated with both orthognathodontic examinations and imaging. Cephalometry, axiography, and electromyography were able to clearly identify the functional impairment but could not give an explanation for the immobility of the left TMJ. Therefore, orthognathodontic evaluation alone would not have been sufficient to formulate a treatment plan.

A more sophisticated investigation of mandibular morphology and masticatory muscles condition was obtained with both CT with 3D reconstruction and MRI of the maxillofacial region. CT showed significant hypoplasia of the left hemimandible (Fig. 1). Both CT and MRI were able to clearly indicate muscular damage on the left side: indeed, the anterior temporalis, masseter, internal and external pterygoid muscles appeared seriously atrophic with almost complete fat substitution (Figs. 2 and 4). Furthermore, maxillofacial MRI confirmed complete immobility of the left condyle (Fig. 3). The articular disk appeared hypoplastic but collocated in a physiological position.

Imaging with CT and MRI made it possible to establish that the left TMJ locking and hypoplasia of the left hemimandible were due to severe muscular pathology, information that was crucial to choose the correct treatment plan. In such conditions, the patient would not have benefited from surgery [17–20]. A more conservative treatment was suggested with functional appliances as function generating bite (FGB) to balance as much as possible the occlusal plane [21–24], knowing that it was not possible to correct the crossbite due to the absence of muscular force on the left side resulting in bone hypoplasia. Some benefits could come from stretching exercises, knowing again that it could not be a resolving therapy [25–28].

## Conclusions

Diagnosis of arthrogryposis is based on clinical examination and molecular studies. Instrumental investigations generally do not show any significant alterations.

Long-term orthopedic management and physiotherapy is required. Physiotherapy soon after birth is helpful in mobilizing joints and preventing disuse atrophy. Sometimes surgical procedures are required.

Treatment of AMC is multidisciplinary and patient-specific, aimed at restoring mobility, ambulation, and the ability to care for oneself. However, in the case of severe maxillofacial involvement, steps should be taken in order to fully investigate the extent of the limitations imposed by the disease on orofacial functions, and to choose the therapeutic means best suited to the specific clinical situation.

This case report highlights the importance of a multidisciplinary approach to the treatment of rare diseases, especially when presenting with unusual features. The treatment of choice should be tailored to the individual patient, after a meticulous diagnostic process. In selected cases, AMC patients with maxillofacial involvement may benefit from conservative orthognathodontic therapy.

## Data Availability

Not applicable.

## Abbreviations

AMC: Arthrogryposis multiplex congenita
CT: Computed tomography
FGB: Function generating bite
MRI: Magnetic resonance imaging
PA: Postero-anterior
SCT: Spiral computed tomography
TMJ: Temporomandibular joint
